# At the threshold of care: Palliative ethics in severe mental illness

**DOI:** 10.1017/S1478951526102326

**Published:** 2026-04-28

**Authors:** Cezar Arruda de Oliveira-Filho, João Carlos Geber-Júnior, Thais Perissotto, Daniel Neves Forte

**Affiliations:** 1 Department of Internal Medicine, Faculty of Medicine of São José do Rio Preto (FAMERP), São José do Rio Preto, Brazil; 2 Faculty of Health Sciences, Sírio-Libanês Hospital, São Paulo, Brazil; 3 Hospital das Clínicas, Faculty of Medicine, University of São Paulo (FMUSP), São Paulo, Brazil

Palliative care has become an increasingly integral component of hospital practice, supported by robust evidence favoring its early integration in life-threatening illness (Kim 2023; Cormack et al. 2025). Its multidimensional approach to suffering – addressing physical, psychological, social, familial, and spiritual domains – offers both transformative potential and substantial clinical complexity (Virdun et al. 2025). Yet palliative care remains a relatively young discipline, and important ethical and clinical thresholds persist, particularly in contexts where established frameworks fail to offer a clear orientation (Denduyver et al. 2025). One such threshold emerges at the intersection of palliative care and severe mental illness.

These gaps become most apparent when palliative principles are applied to patients whose illness primarily affects cognition, communication, and decisional capacity rather than motor function or biological prognosis. In such settings, uncertainty is not an exception but the defining condition of care. It was within this landscape – where proportionality, prognosis, and patient values could not be readily established – that our team encountered the following case, which would challenge foundational assumptions about end-of-life care.

These tensions are not merely theoretical. They emerge at the bedside, often before they can be clearly articulated, in moments when uncertainty itself becomes the central clinical fact.

A man in his early fifties, diagnosed with schizophrenia since early adulthood, was admitted for acute clinical deterioration. He had strong family support, with multiple siblings actively involved in his care. Psychiatric follow-up throughout his life had been intermittent, marked by prolonged periods of medication discontinuation related to poor adherence. Although motor function had remained relatively preserved, his cognitive capacity and interpersonal engagement had progressively declined. Clinicians debated whether this trajectory reflected poorly controlled schizophrenia or the onset of a neurodegenerative process, particularly given that clozapine had never been attempted (Robinson and Holloway 2017).

He had a history of multiple hospital admissions for psychomotor agitation. In the weeks preceding admission, however, his family noted a sudden and marked deterioration in communication, gait instability, and muscular rigidity – features not previously observed. He was admitted for diagnostic investigation and, over the following days, became increasingly rigid, withdrawn, and resistant to interaction.

When first evaluated by our team, he exhibited pronounced muscular rigidity and active resistance to passive movement. Catatonia emerged as the leading diagnostic hypothesis – a clinical diagnosis lacking specific biological markers, characterized by motor, behavioral, and affective disturbances. Benzodiazepines are described as first-line therapy, with electroconvulsive therapy remaining the most established intervention in refractory cases. Prognosis, however, is highly variable, with mortality reported to approach 80% in severe presentations (Hirjak et al. 2024).

Before a comprehensive psychiatric assessment could be completed, the patient experienced rapid clinical deterioration and required admission to the intensive care unit. At this point, the ethical and clinical tension that would define his hospitalization became evident.

Palliative decision-making at the bedside is traditionally structured around a triad (Levy et al. 2012). The first axis concerns functionality, often assessed through motor independence and activities of daily living and operationalized by instruments such as the Palliative Performance Scale or the Karnofsky Performance Status (Osman et al. 2018). The second axis relates to disease trajectory and prognosis, encompassing estimated survival and anticipated response to treatment. The third, and frequently most subjective, axis involves patient values and personal biography, accessed through attentive listening that extends beyond biomedical data.

The case described illustrates what occurs when this triad cannot be fully constructed. How should proportionality be defined under such circumstances? Where should ethical limits be drawn? These questions become particularly acute in the context of severe mental illness (Yıldız 2025).

Severe mental illness includes conditions such as schizophrenia, bipolar disorder, delusional disorders, eating disorders, and severe psychiatric complications such as catatonia and neuroleptic malignant syndrome. In certain refractory cases, major depressive disorder and obsessive–compulsive disorder may also reach comparable levels of severity (Yıldız 2025).

Applying palliative principles in this population reveals immediate tensions. Individuals with chronic schizophrenia or bipolar disorder frequently experience profound impairments in communication, thought organization, and relational engagement. These deficits compromise anamnesis and render the identification of personal values especially challenging (Chan et al. 2023). In such contexts, communication itself becomes the limiting factor – not merely the transmission of information, but the relational conditions required for meaning-making and shared understanding (Geber-Junior and Forte 2025b). At the same time, many retain preserved motor function and independence in basic activities of daily living. As a result, patients may score within normal ranges on functional scales while lacking autonomy in any meaningful existential or relational sense (Foti 2003).

The prognostic axis is no less unstable. Psychiatric illnesses follow highly variable trajectories, and although some may evolve toward neurodegenerative-like syndromes, robust prognostic markers remain scarce. This uncertainty reflects the multiplicity of confounding biological, social, and treatment-related factors inherent to severe mental illness (Coffey et al. 2025).

Within this context, proportional therapeutic planning becomes particularly difficult during acute psychiatric crises or severe medical intercurrences such as catatonia. In the present case, chronic schizophrenia, severe catatonia, and a potentially reversible medical complication made it impossible to determine whether the situation represented a transient decompensation or a terminal event. The patient’s values were inaccessible, and the literature offered no reliable prognostic anchor. The absence of such an anchor is not a failure of medicine, but a recurring condition of palliative practice – particularly when illness trajectories resist linear prediction and demand presence rather than certainty (Geber-Junior and Forte 2025a).

In the face of this uncertainty, the prevailing tendency was to pursue full life-sustaining treatment, including electroconvulsive therapy under mechanical ventilation. Yet an unspoken ethical concern persisted: was this proportional, or an expression of clinicians’ discomfort with uncertainty and fear of dysthanasia (Moureau et al. 2025)?

This uncertainty was shared openly with the family, who remained present throughout the hospitalization. In the absence of guiding frameworks, the decision was made to maintain advanced life-support measures.

Although this case is singular, it reflects a recurrent dilemma encountered in clinical practice. Mortality among individuals with schizophrenia substantially exceeds that of the general population (Jones et al. 2008; Elie et al. 2018). These patients are less likely to receive timely diagnoses, less likely to undergo definitive oncologic treatment, and less likely to receive adequate symptom control near the end of life (Rocque and Cleary 2013). Contrary to common assumptions, most do not die from suicide or violence, but from chronic medical illnesses, including cancer, cardiovascular disease, respiratory illness, and dementia (Foti 2003). Life expectancy is markedly reduced, and professional presence often diminishes in the final months of life (Chochinov et al. 2012).

When appropriately engaged, however, individuals with severe mental illness express concerns indistinguishable from those of the general population: fear of pain, fear of abandonment, and a desire for dignity (Sweers et al. 2013).

Between neglect and dysthanasia lies a narrow ethical threshold. In practice, proportionality is often calibrated not according to patient values, but according to clinicians’ tolerance for uncertainty (Candilis et al. 2004).

Palliative care in the context of severe mental illness still lacks consolidated guidelines. What is already clear, however, is the need for early integration, freedom from prejudice, and interdisciplinary care supported by refined communication skills (Shalev et al. 2020). Patients with severe mental illness do have values; what remains insufficient are the tools required to access them.

Returning to the case, full treatment was maintained. Several sessions of electroconvulsive therapy were performed during the intensive care stay. Weeks later, the patient was encountered again in the outpatient setting. He had not only recovered from pneumonia but demonstrated marked improvement in his psychiatric condition. Communication, autonomy, and functional capacity had returned, surpassing his prior baseline. In retrospect, the apparent cognitive decline likely reflected uncontrolled psychiatric illness rather than irreversible neurodegeneration.

This case offers no definitive answers. It does, however, bring into focus the ethical threshold at which palliative care must often operate in the context of severe mental illness – a space where prognosis is unstable, values are difficult to access, and proportionality cannot be securely anchored. Standing at this threshold requires tolerance of uncertainty, attentiveness to relational presence, and moral humility. Until palliative care develops tools capable of engaging these realities without retreating into either abandonment or excess, care for patients with severe mental illness will continue to unfold at the margins – where ethics is less a matter of resolution than of sustained moral presence.

## References

[ref1] Candilis PJ, Foti MEG and Holzer JC (2004) End-of-life care and mental illness: A model for community psychiatry and beyond. *Community Mental Health Journal* 40, 487–500. doi:10.1023/B:COMH.0000015214.24404.cc 15077725

[ref2] Chan KY, Yap DYH and Singh HGG (2023) Rethinking palliative care in psychiatry. *JAMA Psychiatry* 80, 1081–1082. doi:10.1001/jamapsychiatry.2023.3306 37703027

[ref3] Chochinov HM, Martens PJ, Prior HJ, et al. (2012) Comparative health care use patterns of people with schizophrenia near the end of life. *Schizophrenia Research* 141, 241–246. doi:10.1016/j.schres.2012.07.028 22910402

[ref4] Coffey M, Lugg-Widger F, Hannigan B, et al. (2025) Severe mental illness and last year of life. *Journal of Mental Health* 34, 549–555. doi:10.1080/09638237.2025.2512306 40455545

[ref5] Cormack CL, Lukács M, Chrastek J, et al. (2025) Advancing palliative care through education. *Journal of Hospice & Palliative Nursing* 27, 1–8. doi:10.1097/njh.0000000000001163 41190857

[ref6] Denduyver J, Detraux J, Weydts J, et al. (2025) End-of-life care for people with severe and persistent mental illness. *European Psychiatry* 68, e49. doi:10.1192/j.eurpsy.2025.2440 40123415 PMC12041735

[ref7] Elie D, Marino A, Torres-Platas SG, et al. (2018) End-of-life care preferences in patients with severe mental illness. *American Journal of Geriatric Psychiatry* 26, 89–97. doi:10.1016/j.jagp.2017.09.018 29066037

[ref8] Foti ME (2003) “Do it your way”: End-of-life care for persons with serious mental illness. *Journal of Palliative Medicine* 6, 829–836. doi:10.1089/109662103768253830 14516513

[ref9] Geber-Junior JC and Forte DN (2025a) Caring with time and despite time: Reflections on prognosis. *Palliative and Supportive Care* 23(e148), 1–2. doi:10.1017/S1478951525100680 PMC13166354 40842356

[ref10] Geber-Junior JC and Forte DN (2025b) The Anatomy of connection: Rethinking communication beyond the script. *Palliative and Supportive Care* 23(e216), 1–2. doi:10.1017/S1478951525101235 PMC13166344 41431264

[ref11] Hirjak D, Rogers JP, Wolf RC, et al. (2024) Catatonia. *Nature Reviews Disease Primers* 10, 49. doi:10.1038/s41572-024-00534-w 39025858

[ref12] Jones S, Howard L and Thornicroft G (2008) Diagnostic overshadowing. *Acta Psychiatrica Scandinavica* 118, 169–171. doi:10.1111/j.1600-0447.2008.01211.x 18699951

[ref13] Kim WM (2023) From evidence-based medicine to patient-centered care. *Korean Journal of Anesthesiology* 76, 265–266. doi:10.4097/kja.23543 37501611 PMC10391079

[ref14] Levy MH, Adolph MD, Back A, et al. (2012) Palliative care. *Journal of the National Comprehensive Cancer Network* 10, 1284–1309. doi:10.6004/jnccn.2012.0132 23054879

[ref15] Moureau L, Verhofstadt M, Van Hoe C, et al. (2025) Mapping ethical issues in severe mental illness. *BMC Medical Ethics* 26, 71. doi:10.1186/s12910-025-01234-0 40457393 PMC12128549

[ref16] Osman H, Shrestha S, Temin S, et al. (2018) Palliative care in the global setting. *Journal of Oncology Practice* 14, 431–436. doi:10.1200/JOP.18.00087 29906210

[ref17] Robinson MT and Holloway RG (2017) Palliative care in neurology. *Mayo Clinic Proceedings* 92, 1592–1601. doi:10.1016/j.mayocp.2017.08.003 28982489

[ref18] Rocque GB and Cleary JF (2013) Palliative care reduces morbidity and mortality in cancer. *Nature Reviews Clinical Oncology* 10, 80–89. doi:10.1038/nrclinonc.2012.211 23247373

[ref19] Shalev D, Fields L and Shapiro PA (2020) End-of-life care in serious mental illness. *Psychosomatics* 61, 428–435. doi:10.1016/j.psym.2020.06.003 32660874 PMC7290196

[ref20] Sweers K, Dierckx de Casterlé B, Detraux J, et al. (2013) End-of-life perspectives of patients with schizophrenia. *Archives of Psychiatric Nursing* 27, 246–252. doi:10.1016/j.apnu.2013.05.003 24070994

[ref21] Virdun C, Jones L, Singh GK, et al. (2025) Improving patient-reported experience. *BMC Palliative Care* 24, 245. doi:10.1186/s12904-025-01883-3 41057840 PMC12506362

[ref22] Yıldız E (2025) Palliative care integration in psychiatric disorders. *BMJ Supportive & Palliative Care* 15, 693–700. doi:10.1136/spcare-2025-005441 40983498

